# Lysine deacetylase inhibition promotes relaxation of arterial tone and C-terminal acetylation of HSPB6 (Hsp20) in vascular smooth muscle cells

**DOI:** 10.1002/phy2.127

**Published:** 2013-11-07

**Authors:** Aiqing Chen, Magdalena Karolczak-Bayatti, Michèle Sweeney, Achim Treumann, Kelly Morrissey, Scott M Ulrich, G Nicholas Europe-Finner, Michael J Taggart

**Affiliations:** 1 Institute of Cellular Medicine, Newcastle UniversityNewcastle upon Tyne, U.K.; 2 Protein and Proteome Analysis Facility (AT), Faculty of Medical Sciences, Newcastle UniversityNewcastle upon Tyne, U.K.; 3 Department of Chemistry (SMU), Ithaca CollegeNew York, NY

**Keywords:** Acetylation, HSPB6, vascular tone

## Abstract

There is increasing interest in establishing the roles that lysine acetylation of non nuclear proteins may exert in modulating cell function. Lysine deacetylase 8 (KDAC8), for example, has been suggested to interact with *α*-actin and control the differentiation of smooth muscle cells. However, a direct role of smooth muscle non nuclear protein acetylation in regulating tone is unresolved. We sought to define the actions of two separate KDAC inhibitors on arterial tone and identify filament-interacting protein targets of acetylation and association with KDAC8. Compound 2 (a specific KDAC8 inhibitor) or Trichostatin A (TSA, a broad-spectrum KDAC inhibitor) inhibited rat arterial contractions induced by phenylephrine (PE) or high potassium solution. In contrast to the predominantly nuclear localization of KDAC1 and KDAC2, KDAC8 was positioned in extranuclear areas of native vascular smooth muscle cells. Several filament-associated proteins identified as putative acetylation targets colocalized with KDAC8 by immunoprecipitation (IP): cortactin, *α*-actin, tropomyosin, HSPB1 (Hsp27) and HSPB6 (Hsp20). Use of anti-acetylated lysine antibodies showed that KDAC inhibition increased acetylation of each protein. A custom-made antibody targeting the C-terminal acetylated lysine of human HSPB6 identified this as a novel target of acetylation that was increased by KDAC inhibition. HSPB6 phosphorylation, a known vasodilatory modification, was concomitantly increased. Interrogation of publicly available mass spectrometry data identified 50 other proteins with an acetylated C-terminal lysine. These novel data, in alliance with other recent studies, alert us to the importance of elucidating the mechanistic links between changes in myofilament-associated protein acetylation, in conjunction with other posttranslational modifications, and the regulation of arterial tone.

## Introduction

Protein posttranslational modification (PTM) of lysine residues by acetylation is increasingly recognized to play a prominent role in modulating cell function including that of the cardiovascular system (Kouzarides 2000; Yuan and Marmorstein 2013). Importantly, however, the functional implications of protein lysine acetylation are not restricted to the well-evinced effects of controlling gene expression via nuclear histone acetylation. There is burgeoning proteomic evidence of lysine acetylation of non nuclear proteins (Kim et al. 2006; Choudhury et al. 2009; Sadoul et al. 2011; Lundby et al. 2012). A recent study reported acetylated lysine residues on >4000 proteins from 16 different organs with the majority of proteins expected to have extranuclear locations including those anticipated to be associated with cytoskeletal/myofilament structures (Lundby et al. 2012). Consequently, this necessitates a role for non nuclear lysine deacetylase (KDAC) and/or lysine acetylase (KAT) activities. Indeed, it is now apparent that KDAC function is not restricted to the nucleus (Hubbert et al. 2002; Gupta et al. 2008; Colussi et al. 2011). Of particular relevance to our interest in the regulation of vascular smooth muscle contractility is the possible role of KDAC8 as it has been reported to bind to smooth muscle *α*-actin (*α*-SMA) and act as a marker of smooth muscle differentiation (Waltregny et al. 2004, 2005). However, a direct role of non nuclear protein acetylation in regulating differentiated vascular smooth muscle tone, or a role for KDAC8 in this process, remains unresolved.

We, therefore, sought to establish the direct effect of the class I/II KDAC inhibitor Trichostatin A (TSA) on contraction of isolated rat arteries and compared this with the specific KDAC8 inhibitor compound 2 (Krennhrubec et al. 2008) and the KAT inhibitor plumbagin (Ravindra et al. 2009; Vasudevarao et al. 2012). Nonvascular cell proteomic studies identified cytoskeletal/myofilamentous proteins as potential targets of acetylation including: *α*-SMA, tropomyosin, cortactin, HSPB1 (Hsp27) and HSPB6 (Hsp20) (Kim et al. 2006; Choudhury et al. 2009; Karolczak-Bayatti et al. 2011). We thus also investigated if KDAC inhibition altered the acetylation of these candidates in vascular smooth muscle. Focusing on one of these proteins, we found that KDAC inhibition altered the site-specific acetylation of vascular HSPB6. Our data have revealed new information about the role of protein lysine acetylation in regulating blood vessel tone including the first identification of site-specific vascular smooth muscle protein acetylation following KDAC inhibition.

## Experimental Procedures

### Tissue and experimental solutions

Fresh aorta or mesenteric arteries were dissected from male Wistar rats that had been killed by terminal CO_2_ anesthesia followed by cervical dislocation according to national guidelines. In some experiments human chorionic plate arteries were isolated from placentas obtained, following written informed consent, from normal pregnant women following delivery at term. The following solutions were used: physiological saline solution (PSS) mmol/L composition: NaCl 119; KCl 4.7; MgSO_4_ 2.4; NaHCO_3_ 25; KH_2_PO_4_ 1.17; Glucose 6.05; EDTA (Ethylene diamine tetraacetic acid) 0.0684; CaCl_2_ 1.6. High potassium solution (KPSS) with composition as per PSS with an isosmotic substitution of NaCl) with KCl (60 mmol/L final concentration). Phenylephrine (PE) hydrochloride and acetylcholine chloride stocks were made up daily in distilled water; TSA stock was made up with ethanol and compound 2 (manufactured as described previously Krennhrubec et al. 2008) and plumbagin in dimethylsulfoxide (DMSO).

### Myography experiments

Aortic rings were suspended in PSS in 10 mL water jacketed organ baths maintained at 37°C, aerated (5% CO_2_, 95% air gas mixture), stretched to reach a resting tension of 2 g and allowed to equilibrate for 30 min. Mesenteric arteries were mounted in a myograph chamber (Danish Myotech, Aarhus, Denmark) and stretched to a normalized internal circumference of 0.9L_100_ where L_100_ is the internal circumference equivalent to a transmural pressure of 100 mmHg. Contractions were induced using either *α*1-adrenergic receptor agonist PE (1 μmol/L) or KPSS and allowed 30 min to reach steady state. Vessels were then exposed to reagents or their respective vehicle control for up to 20 min. Force was expressed as absolute values (mN/mm). Statistical evaluation was carried out using one- or two-way analysis of variance (ANOVA) to compare the curves, followed by Bonferroni post tests. The data were considered to be statistically significant where *P* < 0.05.

### Immunofluorescent microscopy

Cryosections (10 μm) of aorta tissue were fixed with acetone for 10 min at room temperature and then kept at −80°C until use for immunofluorescence (IF) studies. After permeabilization with 0.1% triton X-100 in TBS (Tris-bufferred saline) (5 mmol/L Tris, 137 mmol/L NaCl, pH 7.6), sections were blocked with 5% bovine serum albumin (BSA) in TBS for 1 h and then incubated with primary antibody in 1% BSA/TBS for another hour. They were then washed with TBS, and incubated with rabbit anti-mouse IgG, or goat anti-rabbit IgG, conjugated to Alexa Fluor 488 or Alexa Fluor 568 (Invitrogen Life Technologies, Paisley, U.K.) for 1 h. Yoyo-1 was used for nuclear counterstaining. Images were observed on an Andor Revolution XD (Belfast, U.K.) confocal microscope system coupled to an iXon EMCCD camera.

### Preparation of vascular protein homogenates

Arterial tissues were manually homogenized in Western blotting (WB) buffer (62.5 mmol/L Tris-Cl pH 6.8, 2% Sodium dodecyl sulfate (SDS), 10% sucrose, protease, and phosphatases inhibitors), and incubated for 30 min on ice, or prepared for Immunoprecipitation (IP)s by homogenization with a Minilys bead machine (Bertin Technologies, Aix-en-Provence, France) in NP-40 buffer (1% NP-40, 50 mmol/L Tris base, 1 mmol/L EDTA, 5% Glycerol, inhibitors as detailed above). Homogenates were centrifuged for 15 min at 14,000 × g at 4°C and supernatants stored at −80°C. Protein concentration was measured using the DC™ protein assay (Bio-Rad, Hemel Hempstead, U.K.).

### Immunoprecipitation assays

Immunoprecipitation was performed as described previously^13^ with 1 mg of protein extracts placed in four volumes of co-IP buffer (20 mmol/L 4-(2-hydroxyethyl)-1 piperazineethanesulfonic acid, pH 7.9, 75 mmol/L KCl, 2.5 mmol/L MgCl_2_, and 0.1% NP-40, protease and phosphatase inhibitors). Proteins were precleared with 2 μg of rabbit IgG (ab46540; Abcam, Cambridge, U.K.) and 20 μL of protein A-coated magnetic beads (10001D; Invitrogen) for 45 min at 4°C. Precleared proteins were incubated with 2 μg of the respective primary antibodies overnight at 4°C. Protein/Ab complexes were recovered with 25 μL of protein A-coated magnetic beads and washed four times with co-IP buffer. Proteins were retrieved by boiling for 5 min in 20 μL of loading buffer (250 mmol/L Tris-Cl pH 6.8, 4% SDS, 10% glycerol, 2% *β*-mercaptoethanol) with fresh 100 mmol/L 1,4-Dithiothreitol and then subjected to WB.

### Western blotting

Protein extracts were electrophoresed in 12% SDS-PAGE, followed by electro-transfer to polyvinylidene difluoride membrane (Bio-Rad). For normal WB, after blocking with 5% fat-free dry milk and 0.1% tween-20 in TBS (TBS-T), the blot was incubated with primary antibodies for 1 h at room temperature or overnight at 4°C. The blots were washed three times in TBS-T, and then incubated (1:200) with goat anti-rabbit or anti-mouse–HRP conjugated secondary antibody (Dako, Ely, U.K.). For IP-WB, membranes were blocked with native staphylococcus aureus protein A (product number 7840-0604; AbD Serotec, Kidlington, U.K.) in TBS-T (2.5 μg protein A/1 mL TBS-T) for 1 h at room temperature, and washed with TBS-T three times before incubated with primary antibodies overnight at 4°C. Blots were further incubated with protein A-conjugated horse radish peroxidase (product number 18–160; Millipore, Watford, U.K.) for 1 h at room temperature and membranes exposed to enhanced chemiluminescence solution (Amersham Pharmacia Biotechnology, Amersham, U.K.). For WB, blocked membranes were incubated with goat anti-rabbit (PO448; Dako) or goat anti-mouse (PO447; Dako) horse radish peroxidase-conjugated secondary antibody. Membranes were exposed to photographic film and developed images densitometrically scanned and digitally stored. Quantification of densitometric scans was performed using Intelligent Quantifier software (Bioimage, Prague, Czech Republic).

### Antibodies

Commercially available primary antibodies used were as follows: anti-KDAC1 (mouse, Millipore 05–100, 1:100 for IF), anti-KDAC2 (mouse, Abcam ab12169, 1:100 for IF), anti-KDAC8 (rabbit, Abcam ab38664, 1:2000 for WB, 1:100 for IF), anti-acetylated histone3 (mouse, anti-Ac-H3, Upstate 06-599, 1:10,000 for WB), anti-HSPB6 (rabbit, Abcam Ab13491, 1:10,000 for WB, 1:100 for IF), anti-HSPB1 (rabbit, Abcam Ab79868, 1:10,000 for WB, 1:100 for IF), anti-cortactin (rabbit, Millipore AB3887, 1:1000 for WB, 1:100 for IF), anti-tropomyosin (mouse, Sigma T2780, 1:1000 for WB, 1:150 for IF), anti-*α*-actin (mouse, Sigma A2547, 1:40,000 for WB, 1:500 for IF), anti-acetylated lysine (rabbit, ImmunoChem, 2ug for IP), anti-phospho-Hsp20 (rabbit, Abcam Ab58522, 1:1000 for IP). Nonimmune IgG (rabbit, Abcam ab46540 or mouse, Millipore 12-371) was used as a negative control for IP experiments.

### Customized antibody production

Separate peptides corresponding to regions adjacent to each of the three lysine residues of human HSPB6 (UniProtKB/Swiss-Prot accession: O14558.2), that encompassed a chemically acetylated version of the requisite lysine residue(s), were used as antigens for polyclonal antibody production. The acetylated peptides and antibodies were produced by Cambridge Research Biochemicals, U.K. Separate rabbits were immunized five times with 1 mg of the chosen acetylated peptide (Freund incomplete adjuvant). The antibodies were affinity-purified against the designated acetylated cognate peptide. The antibody specificity was tested in Western dot blot analysis at 1:3000 dilution against 2 μg of the acetylated peptide and compared to signal generated against 2 μg of non acetylated version of the chosen peptide. Verified anti-Ac-human HSPB6 antibody was subsequently used to determine the extent of acetylated HSPB6 in control and untreated tissues of human placental arteries.

## Results

### KDAC inhibition induces vascular smooth muscle relaxation

**Figure 1:**
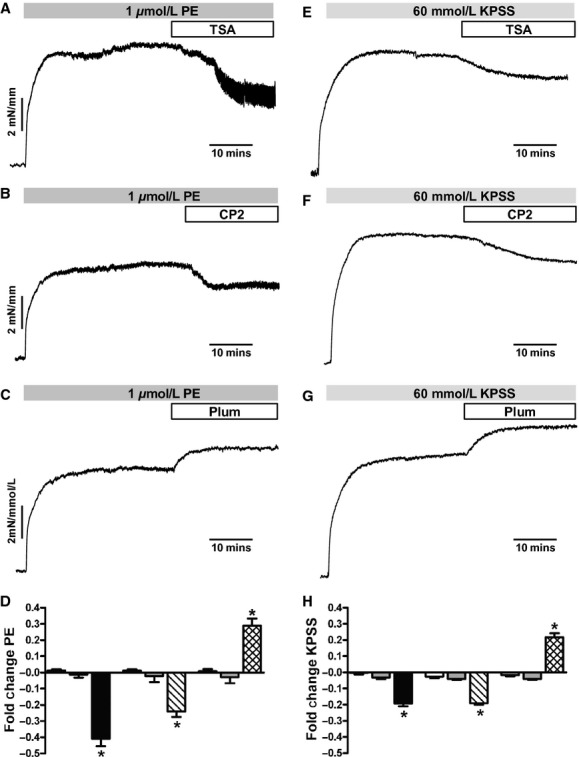
KDAC inhibition results in reduced arterial constriction. Aorta segments preconstricted with1 μmol/L phenylephrine (A–C) or 60 mmol/L KPSS (E–G) relaxed upon exposure to the KDAC inhibitors TSA (3 μmol/L) or compound 2 (200 μmol/L) but constricted to the KAT inhibitor plumbagin (2.5 μmol/L). The bar charts (D, H) exhibit maximum changes in tension with time controls (white), vehicle controls (gray), TSA (black), compound 2 (CP2, hatched lines) or plumbagin (cross-hatched bars). *different from corresponding time and vehicle controls.

Preconstricted aorta segments were exposed to the class I/II KDAC inhibitor TSA or the KDAC8 inhibitor compound 2 (Fig. 1). TSA relaxed PE or KPSS preconstricted vessels by 41.1 ± 3.8% (*n* = 11) and 19.3 ± 0.1% (*n* = 11), respectively; relaxations to compound 2 were 24.1 ± 1.9% (*n* = 9) and 19.1 ± 0.1% (*n* = 11). Similar results were obtained with mesenteric arteries preconstricted with KPSS wherein TSA or compound 2 relaxed vessels by 22.2 ± 2.8% and 20.4 ± 2.6%, respectively.

In contrast, the KAT inhibitor plumbagin, which inhibits KAT3B/3A (also known as p300/CREB-binding protein), resulted in an increase in tone of 28.8 ± 0.04% and 21.6 ± 0.02% of arteries preconstricted, respectively, with PE or KPSS (Fig. 1).

### KDAC localization in native aortic tissue

**Figure 2:**
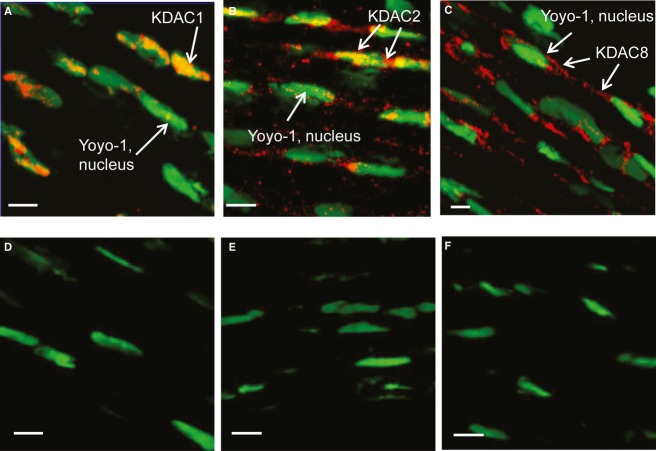
Class I KDAC localization in arterial smooth muscle. (A) KDAC1 (colored red), is located exclusively in the nucleus (colored green) of aorta smooth muscle cells; nuclear colocalization is indicated by yellow coloring. (B) KDAC2 (colored red), is not only located in predominantlynuclear (green) areas of the cell but also in some non nuclear areas. (C) KDAC8 (red) is localized exclusively to areas distinct from the nucleus (green). (D–F) represent the respective no primary antibody control images. Scale bar = 10 μm.

KDAC8 has previously been shown to have a distribution outside of the nucleus in smooth muscle cells (Waltregny et al. 2004). Given the above acute actions of KDAC inhibitors on vascular tone it was of interest to establish the localization of KDAC8 in native, contractile aorta smooth muscle tissues compared to that of other class I KDACs. Although KDAC1 was localized to the nucleus, and KDAC2 showed both nuclear and extranuclear localization, KDAC8 was expressed exclusively in non nuclear areas in smooth muscle cells of aorta tissue (Fig. 2).

### Possible non nuclear targets of KDAC8 inhibition

**Figure 3:**
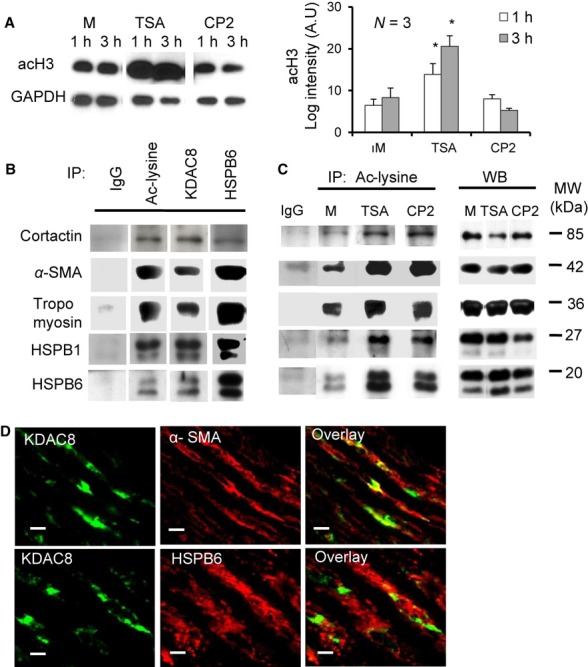
Protein acetylation following KDAC inhibition. (A) TSA increased the level of nuclear acetylated histone 3 (acH3) after 1 or 3 h (*n* = 3 separate experiments). This was not seen with the KDAC8 inhibitor compound 2 (CP2) nor with medium (M) control. GAPDH served as a loading control. (B) After immunoprecipitation (IP) with antibodies against acetylated lysine (Ac-lysine), KDAC8 or HSPB6, lysates were probed for the myofilament-associated proteins cortactin, *α*-smooth muscle actin (SMA), tropomyosin, HSPB1 or HSPB6. Each immunoprecipitating antibody pulled down each protein of interest in a manner not seen with non immune IgG (IgG) serving as a negative control. (C) TSA or compound 2 (CP2) treatment qualitatively increased the levels of immunoprecipitated acetylated cortactin, *α*-SMA, tropomyosin, HSPB1 and HSPB6 compared to medium alone. Western blotting (WB) was used to check that the individual protein expression levels following treatment were unchanged. (D) Immunofluorescent staining indicates KDAC8 colocalization in aorta smooth muscle cells with areas of *α*-smooth muscle actin (SMA) or HSPB6-positive staining.

The above localization data suggested that KDAC8 would be unlikely to regulate nuclear protein acetylation whereas TSA, as an inhibitor all class I/II KDACs including the nuclear-resident KDAC1 and KDAC2, would be expected to do so. Indeed, TSA treatment increased nuclear histone3 protein acetylation (as indicated by an anti-Ac-H3 antibody), yet even prolonged exposure up to 24 h to compound 2 treatment was without effect (Fig. 3A). Similar timed exposure to medium-only or diluent controls (for TSA or compound 2) also had no effect. Additional support to the notion that *α*-SMA, cortactin, tropomyosin, HSPB6, and HSPB1 were possible targets for KDAC8 interaction was established by co-IP of each protein with anti-KDAC8 antibody but not, importantly, with control nonimmune IgG (Fig. 3B). In addition, each protein was immunoprecipitated by anti-acetylated lysine antibodies (Fig. 3C). Moreover, TSA or compound 2 treatment appeared to increase the amount of each protein immunoprecipitated by anti-acetylated lysine antibody and the right hand side of Figure 3C (WB) indicates that this occurred without a change in individual total protein content indicative of an increase in protein acetylation following KDAC inhibition. Immunofluorescent costaining of KDAC8 with either *α*-SMA or HSPB6 indicated colocalization albeit the distribution of KDAC8 was more restricted than that of *α*-SMA or HSPB6.

### C-terminal lysine acetylation of HSPB6

**Figure 4:**
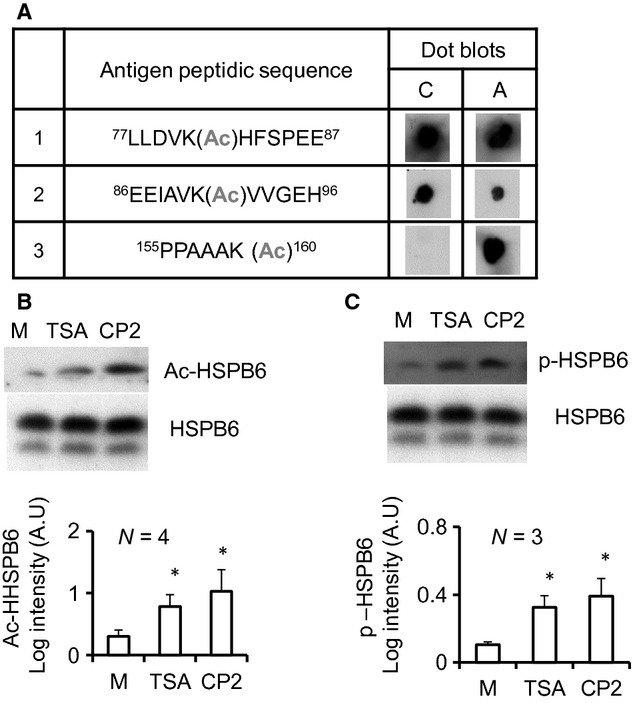
KDAC inhibition increases c-terminal acetylation of HSPB6 and phosphorylation of Ser 16. (A) Dot blot assay of the peptide sequences used as antigens for acetylated HSPB6 antibodies indicated the response of respective antibodies to control (non acetylated) and acetylated peptides. (B) Antibody directed against acetylated c-terminal HSPB6 indicated an increased acetylation of HSPB6 following TSA or compound 2 (CP2) treatment. (C) TSA or compound 2 (CP2) also increased phosphorylation of HSPB6 Ser^16^. *indicates significant difference from medium control (M).

Of these proteins examined, HSPB6 is known to contain three lysine residues. Peptides derived from regions adjacent to these residues, each containing an acetylated form of the lysine, were prepared as immunogens for antibody production. Only the antibody directed against the C-terminal acetylated lysine residue showed specificity of binding to the respective purified acetylated peptides (Fig. 4A). Use of this antibody established that HSPB6 C-terminal lysine acetylation was increased by TSA (to 2.26-fold ± 0.19 above control) and compound 2 (to 3.02-fold ± 0.34) (Fig. 4B). These changes occurred without any alteration in individual protein content (Fig. 4B). When probing tissue lysates for changes in HSPB6 ser^16^ phosphorylation (Fig. 4C) this was also found to be elevated by TSA or compound 2 treatment.

### Bioinformatic identification of other c-terminally acetylated proteins

**Figure 5:**
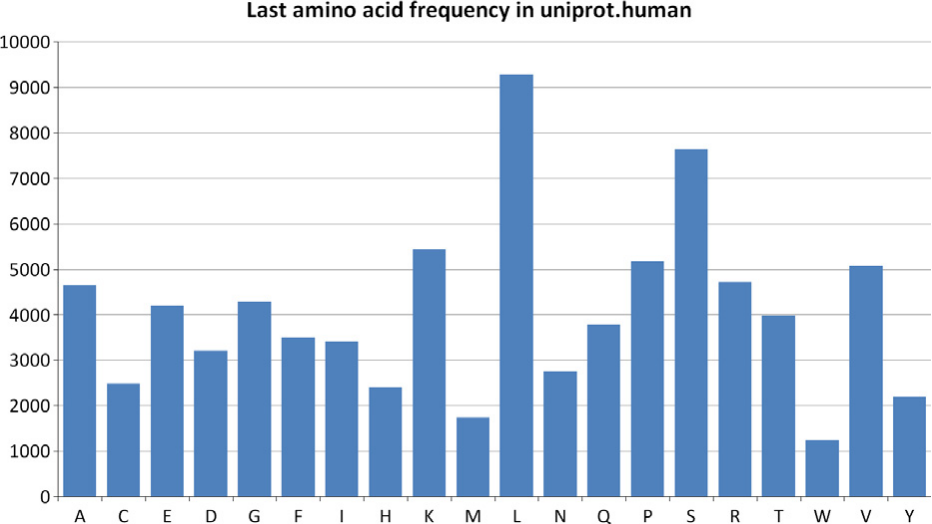
Last aa frequency in the http://www.uniprot.org database.

Publicly available protein databases (http://www.uniprot.org) indicate that over 5000 protein entries have a c-terminal lysine residue (Fig. 5). It was of interest, therefore, to consider if the novel c-terminal lysine acetylation we identified for HSPB6 may be indicative of a broader regulatory modality covering other proteins. The mass spectrometric data of Lundby et al. (2012) examined the acetylated proteome from 16 different rat organs isolated under basal conditions. We interrogated this dataset further for evidence of c-terminally acetylated lysine residues and identified 50 proteins with a positive indication of this modification (Fig. 5 and Table 1).

**Table 1:** Identification of c-terminal acetylated lysine residues from mass spectrometric datasets

Leading protein	Uniprot	Protein descriptions
IPI00111255.1	P17665	Cytochrome c oxidase subunit 7C, mitochondrial precursor; 7 kDa protein; hypothetical protein isoform 1; hypothetical protein
IPI00117312.1	P05202	Aspartate aminotransferase, mitochondrial precursor
IPI00119220.1	P62317; Q14AF6	Small nuclear ribonucleoprotein Sm D2; hypothetical protein LOC680309; similar to Sm D2
IPI00121419.1		hypothetical protein
IPI00121534.11	P00920	Carbonic anhydrase 2
IPI00127596.1	P07310; A2RTA0; Q9D6U7;P00564	Creatine kinase M-type; Creatine kinase M-type
IPI00133985.1	P60122; Q3U1C2; P60123;Q3UJN2	RuvB-like 1; RuvB-like 1; CRL-1722 L5178Y-R cDNA, RIKEN full-length enriched library, clone: I730064L05 product: RuvB-like protein 1, full insert sequence
IPI00188162.3	Q9D4A5	hypothetical protein LOC498943; hypothetical protein LOC71046
IPI00190377.2	Q9EQS0; Q93092	Transaldolase; Transaldolase
IPI00191794.2	P18437	Non-histone chromosomal protein HMG-17
IPI00192188.4	P63255; P63254; Q6P3B4; Q8C2N7	Cysteine-rich protein 1; Cysteine-rich protein 1
IPI00194222.1	P10888	Cytochrome c oxidase subunit 4 isoform 1, mitochondrial precursor
IPI00201307.1	Q9CQB4;Q9D855	similar to ubiquinol-cytochrome c reductase binding protein; similar to ubiquinol-cytochrome c reductase binding protein; 11 kDa protein; Adult male cerebellum cDNA, RIKEN full-length enriched library, clone: 1500015I13 product: UBIQUINOL-CYTOCHROME C REDUCTASE
IPI00201586.1	P42930; P14602-1; P14602; Q545F4; P14602-2; P14602-3	Heat shock protein beta-1; Isoform A of Heat shock protein beta-1; Isoform B of Heat shock protein beta-1; 19 kDa protein; Isoform C of Heat shock protein beta-1; 17 kDa protein
IPI00202616.1	Q9DCT2	NADH dehydrogenase (ubiquinone) Fe-S protein 3; NADH dehydrogenase [ubiquinone] iron-sulfur protein 3, mitochondrial precursor; similar to NADH dehydrogenase (ubiquinone) Fe-S protein 3
IPI00202842.1	Q9D735	hypothetical protein LOC288920; 11 kDa protein; Uncharacterized protein C19 or f43 homolog
IPI00203829.1	Q9WUC4; O08997; Q5NCU2	Copper transport protein ATOX1; Copper transport protein ATOX1
IPI00209347.2	Q5XIX3; Q8K4J0-1; Q8K4J0; A2AJG6; Q8K4J0-2; A2AJG7; Q3TLT6; A2AJG5; A2AJG9; Q32MX8; Q8K4J0-3; A2AJG8	Artemis protein; Isoform 1 of Artemis protein; 78 kDa protein; Isoform 2 of Artemis protein; Mammary gland RCB-0526 Jyg-MC(A) cDNA, RIKEN full-length enriched library, clone: G830039E04 product: DNA cross-link repair 1C, PSO2 homolog (S. cerevisiae), full insert
IPI00210071.3	Q91ZN1; O89053; Q3T9L1; Q3U1N0; Q3U232; Q3U9K3	Coronin-1A; Coronin-1A; Bone marrow macrophage cDNA, RIKEN full-length enriched library, clone: I830036K05 product: coronin, actin binding protein 1A, full insert sequence
IPI00210158.1	Q63570; Q3TFA5; Q3TJ97; Q3TUN5; Q3UBF0; Q52L53; Q569X4; Q6ZWN9; Q8BKU2; Q8K3E0; P54775	26S protease regulatory subunit 6B; proteasome 26S ATPase subunit 4; 26S protease regulatory subunit 6B
IPI00210920.1	P00507	Aspartate aminotransferase, mitochondrial precursor
IPI00211206.7	P52944; O70400; Q3TZ17	PDZ and LIM domain protein 1; PDZ and LIM domain protein 1
IPI00211593.1	P07895	Superoxide dismutase (Mn), mitochondrial precursor
IPI00228748.1	Q8CD94	Protein lin-52 homolog; similar to lin-52 CG15929-PA
IPI00230787.5	P27139	Carbonic anhydrase 2
IPI00230832.7	P11951; Q78EE8; Q9Z1G9	Cytochrome c oxidase polypeptide VIc-2
IPI00230937.5	P31044	Phosphatidylethanolamine-binding protein 1
IPI00230942.5	P08009; Q3TRV7; Q3V4E2; Q6PJ91; Q80W21	Glutathione S-transferase Yb-3; 12 days embryo embryonic body between diaphragm region and neck cDNA, RIKEN full-length enriched library, clone: 9430034P22 product: Glutathione S-transferase Yb-3 (EC 2.5.1.18) (Chain 4) (GST Yb3) (GST class-mu 3) homolog; 25 k
IPI00231028.2	P56571	ES1 protein homolog, mitochondrial precursor
IPI00231611.7	P14408-1; P14408	Isoform Mitochondrial of Fumarate hydratase, mitochondrial precursor
IPI00231978.5	P29419; Q06185; Q5EBI8	ATP synthase subunit e, mitochondrial; ATP synthase e chain, mitochondrial
IPI00339996.7		21 kDa protein
IPI00352475.3	Q3UHX2; A0JLS1; Q1WWJ8; Q62785	28 kDa heat- and acid-stable phosphoprotein; 28 kDa heat- and acid-stable phosphoprotein
IPI00358872.2	Q5XI85; Q8CFA2; A2RSW6	Aminomethyltransferase; Aminomethyltransferase, mitochondrial precursor
IPI00365904.4	Q80Y14; Q3UF85	glutaredoxin 5 homolog; Glutaredoxin-related protein 5
IPI00366416.2	Q9D0M3-1;Q9D0M3;Q9D0M3-2	similar to cytochrome c-1; Isoform 1 of Cytochrome c1, heme protein, mitochondrial precursor; Isoform 2 of Cytochrome c1, heme protein, mitochondrial precursor; similar to cytochrome c-1
IPI00367259.2	Q5M949	Nipsnap homolog 3A; similar to NIPSNAP-related protein isoform 2; similar to NIPSNAP-related protein isoform 1
IPI00368304.2	Q99PP7-1; Q99PP7; Q99PP7-2	similar to tripartite motif protein 33; similar to tripartite motif protein 33 isoform 2; similar to tripartite motif protein 33 isoform 1; similar to tripartite motif protein 33 isoform 3; Isoform Alpha of E3 ubiquitin-protein ligase TRIM33; Isoform Beta of E3
IPI00373418.3	Q99PU6; P53395; Q3TMF5; Q6LC11; Q7TND9	dihydrolipoamide branched chain transacylase E2; Lipoamide acyltransferase component of branched-chain alpha-keto acid dehydrogenase complex, mitochondrial precursor
IPI00387284.5	Q3URR2; Q921S6; O89038; Q66HL8; Q63943-1; Q63943; Q63943-2	Myocyte enhancer factor 2D; MEF2D protein; Isoform Non-muscle of Myocyte-specific enhancer factor 2D; Isoform Muscle of Myocyte-specific enhancer factor 2D; 54 kDa protein
IPI00389571.6	Q10758; Q6LCB1	Keratin, type II cytoskeletal 8
IPI00411230.3	P08010	Glutathione S-transferase Mu 2; 17 kDa protein
IPI00555265.1	Q5BJZ3; Q61941; Q3TGH1; Q3TWH2; Q8BGK0; Q8C1W8; Q922E1; Q8C3H2; Q8C9V5; Q8C337	Nicotinamide nucleotide transhydrogenase; NAD(P) transhydrogenase, mitochondrial precursor; nicotinamide nucleotide transhydrogenase; 76 kDa protein; 116 kDa protein; 116 kDa protein; 16 days neonate heart cDNA, RIKEN full-length enriched library, clone: D830027D
IPI00627078.1	Q4KLJ0;P09602;Q58E57; Q5BL14; A3KGL9; Q5XK38	High mobility group nucleosomal binding domain 2; Non-histone chromosomal protein HMG-17; similar to put. HMG-17 protein; High mobility group nucleosomal binding domain 2; Hmgn2 protein; hypothetical protein
IPI00654464.2	Q3KRE2; Q3SWU1	Methyltransferase like 7A
IPI00764111.1	Q6A077; Q8BK95; Q64331	similar to Myosin-6; MKIAA0389 protein; 146 kDa protein; myosin VI;0 day neonate eyeball cDNA, RIKEN full-length enriched library, clone: E130318C17 product: MYOSIN VI homolog; similar to Myosin-6; 146 kDa protein; Myosin-VI
IPI00765431.1	P50136; Q3U3J1; Q99L69; P11960; Q5EB89	similar to 2-oxoisovalerate dehydrogenase alpha subunit, mitochondrial precursor; NOD-derived CD11c +ve dendritic cells cDNA, RIKEN full-length enriched library, clone: F630105A02 product: branched chain ketoacid dehydrogenase E1, alpha polypeptide, full inse
IPI00767147.1	P04764; Q5BJ93; Q5EB49; P17182; Q5FW97; Q6PHC1	similar to Alpha-enolase; Alpha-enolase; Alpha-enolase; Enolase
IPI00778559.1	Q66HT2; Q8BU11; Q3U661; Q3UGN7; Q3UVQ1; Q6A006; Q99PM1	Epidermal Langerhans cell protein LCP1; TOX high mobility group box family member 4; TOX high mobility group box family member 4
IPI00870114.1		glutaryl-Coenzyme A dehydrogenase

The mass spectrometry datasets of Lundby et al. (2012) were assessed as follows: the position of the last acetylated lysine was extracted from all quantified acetylated lysine residues using column C of the Excel workbook of their datset mmc3.xls (http://www.sciencedirect.com/science/MiamiMultiMediaURL/1-s2.0-S2211124712002161/1-s2.0-S2211124712002161-mmc3.xls/280959/FULL/S2211124712002161/1baaef15a6f6473f5ddba2caa916046b/mmc3.xls). This number was identical to the sequence length of the protein (column P in the same table) for 50 proteins, which are listed in the present table.

## Discussion

We identify the novel acetylation of non nuclear, KDAC8-interacting myofilament-associated proteins as a mechanism for modulating vascular smooth muscle contractility. KDAC inhibition results in vasorelaxation and an increased acetylation of several myofilamentous proteins. In contrast, KAT inhibition with plumbagin resulted in increased tone; thus, these data point to a likely role for myofilament protein acetylation determining contractile function. In particular, we have provided evidence pointing to a key role for KDAC8 and the acetylation of the c-terminal lysine residue of HSPB6 in this process.

Co-IP with several filament-interacting proteins, and co-immunofluorescent staining with *α*-actin and HSPB6, suggested a prominent role for KDAC8 in mediating the relaxatory actions of KDAC inhibitors. Indeed, compound 2 increased the amount of cortactin, tropomyosin, *α*-actin, HSPB1 and HSPB6 immunoprecipitated with anti-lysine antibodies. It is intriguing to note, however, that KDAC8 localization is more restricted than that of *α*-actin and HSPB6 perhaps indicating why KDAC inhibition does not completely relax tone. Notably, an acetylation prediction tool (http://www.phosida.com) identified cortactin, tropomyosin, *α*-actin and HSPB1 to contain at least one internal lysine residue as a putative acetylation site.

The investigation of particular lysine residues targetted by acetylation is hampered by a paucity of efficacious, commercially available acetylation site-specific antibodies. HSPB6, an actin filament-interacting protein known to influence vascular smooth muscle relaxation via Ser^16^ phosphorylation (Rembold et al. 2001), contains three lysines. Our attempt to raise antibodies to peptide regions encompassing each of these three lysines, as acetylated forms, was successful only for the c-terminal lysine. This antibody detected an increase in acetylated HSPB6 in response to KDAC inhibition with compound 2 or TSA. This complements our recent finding that nonvascular smooth muscle HSPB6 acetylation accompanies KDAC inhibition (Karolczak-Bayatti et al. 2011). Over 5000 UniProt-listed proteins (http://www.uniprot.org) end with a lysine residue and our examination of a recent large-scale proteomic dataset identified 50 proteins with an acetylated modification in this residue. Thus, c-terminal protein lysine acetylation that we have identified herein for HSPB6 is likely to be part of a more general mechanism for regulating protein and cell function.

Notably, each of the proteins identified herein participate in regulating myofilament dynamics. Moreover, it is probable that these proteins form but a small part of the full-range of vascular contractile-associated proteins with the potential to be regulated by acetylation (Kim et al. 2006; Choudhury et al. 2009; Lundby et al. 2012). In particular, increased HSPB6 phosphorylation accompanied KDAC inhibition pointing to a potential synergistic vasodilatory influence of c-terminal lysine acetylation and ser^16^ phosphorylation (Rembold et al. 2001). The mechanism of the increased ser^16^ phosphorylation upon KDAC inhibition remains to be elucidated. HSPB6 is phosphorylated at this site by protein kinase A (PKA) or Protein kinase G (PKG) and so, one possibility, is that acetylation of these kinases (following KDAC inhibition), or proteins that interact with these kinases, increases their activity toward HSPB6. Certainly, in reverse, PKA/PKG stimulation is known to phosphorylate and alter KDAC (Lee et al. 2004) and KAT activities (Colussi et al. 2012) lending support to the general notion that acetylation and phosphorylation may be PTMs that often operate in tandem to determine protein and cell function. In particular, PKA-mediated phosphorylation HSPB6 is associated with vasodilation and phosphorylation of KDAC8 inhibits its activity which, one presumes, may also give a relaxatory influence akin to that seen here with direct KDAC inhibition. It will be of interest to explore these molecular mechanisms in more detail in future work for example, to examine the combinatorial actions of forskolin (to inhibit PKA) and KDAC inhibitors. Additionally, the extent of relaxation upon KDAC inhibition with TSA was greater for arteries preconstricted with PE than high potassium depolarizing solution suggesting that part of the dilatory influence may be due to an effect on membrane potential.

A number of recent reports postulate roles for other KDAC isoforms in mediating vascular smooth muscle cell proliferation, differentiation, and function particularly via regulation of transcriptional events (Mattagajasingh et al. 2007; Zhou et al. 2011). Collectively, these data contribute to an increasing appreciation that vascular function is influenced by protein acetylation and emphasizes the need, in future work, to characterize the vascular cell- and KDAC-specific acetylomes in order to understand the complexity of tone regulation by this PTM.

In conclusion, we provide evidence to implicate a novel role for acetylation of vascular smooth muscle non nuclear proteins in regulating arterial activity and highlight important roles for KDAC8 and HSPB6 in this process. This, and complimentary recent studies (Colussi et al. 2012), draw attention to myofilament-associated protein acetylation as an attractive feature for potential targeting by current (Jung et al. 2010; Colussi et al. 2012) or future therapies for vascular pathophysiologies.
